# Supplementary material for: Large-scale predictions of alternative protein conformations by AlphaFold2-based sequence association
Source: Nat Commun. 2025 Jul 1;16:5622. doi: 10.1038/s41467-025-60759-5 (PMC12219334; doi:10.1038/s41467-025-60759-5)
Supplement: Supplementary file 1 — Supplementary Information [file 41467_2025_60759_MOESM1_ESM.pdf]

Supplementary Information for

Large-scale predictions of alternative protein conformations by AlphaFold2-based sequence association

Myeongsang Lee[1,†], Joseph W. Schafer[1,†], Jeshuwin Prabakaran[1], Devlina Chakravarty[1], Madeleine F. Clore[1], and Lauren L. Porter[1,2,*]

[1]National Center for Biotechnology Information, National Library of Medicine, National Institutes of Health, Bethesda, MD 20894, USA
[2]Biochemistry and Biophysics Center, National Heart, Lung, and Blood Institute, National Institutes of Health, Bethesda, MD, 20892, USA
[†]Contributed equally to this work

*Correspondence: porterll@nih.gov

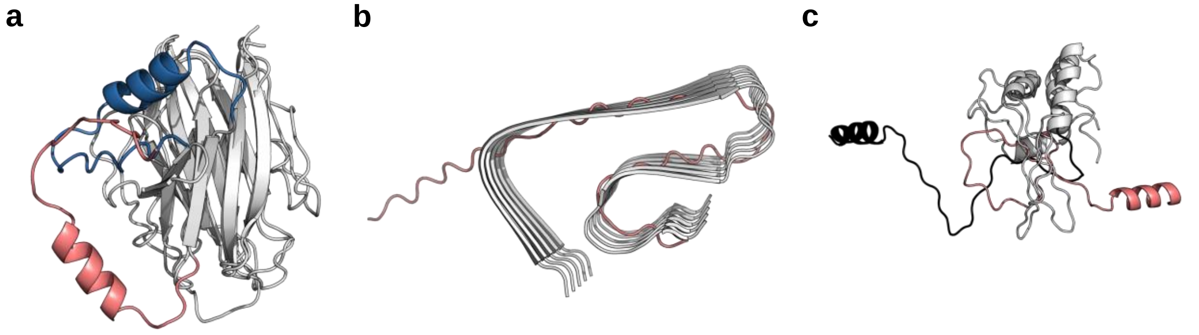

Supplementary Figure 1. CF-random predictions resemble monomeric units in assemblies, suggesting that the AF2 multimer weights may provide more context and improve predictions of alternative conformations. (a). The full MSA prediction of FraC has its helix nicely packed against the rest of the protein (blue), whereas sampling at shallow depth (2:4) leads to a detached helix more like the multimeric pore form (pink). (b). Sampling at shallow depth (1:2) produces an Aβ42 monomer (pink) resembling on a fibril in the PDB (gray, PDB ID: 5oqv). (c). Single sequence predictions of yeast Cks1 produce an unfolded C-terminal region (pink) similar to that observed in its experimentally determined domain-swapped dimer (black, PDB ID: 1qb3). Source data are included in the Source Data file.

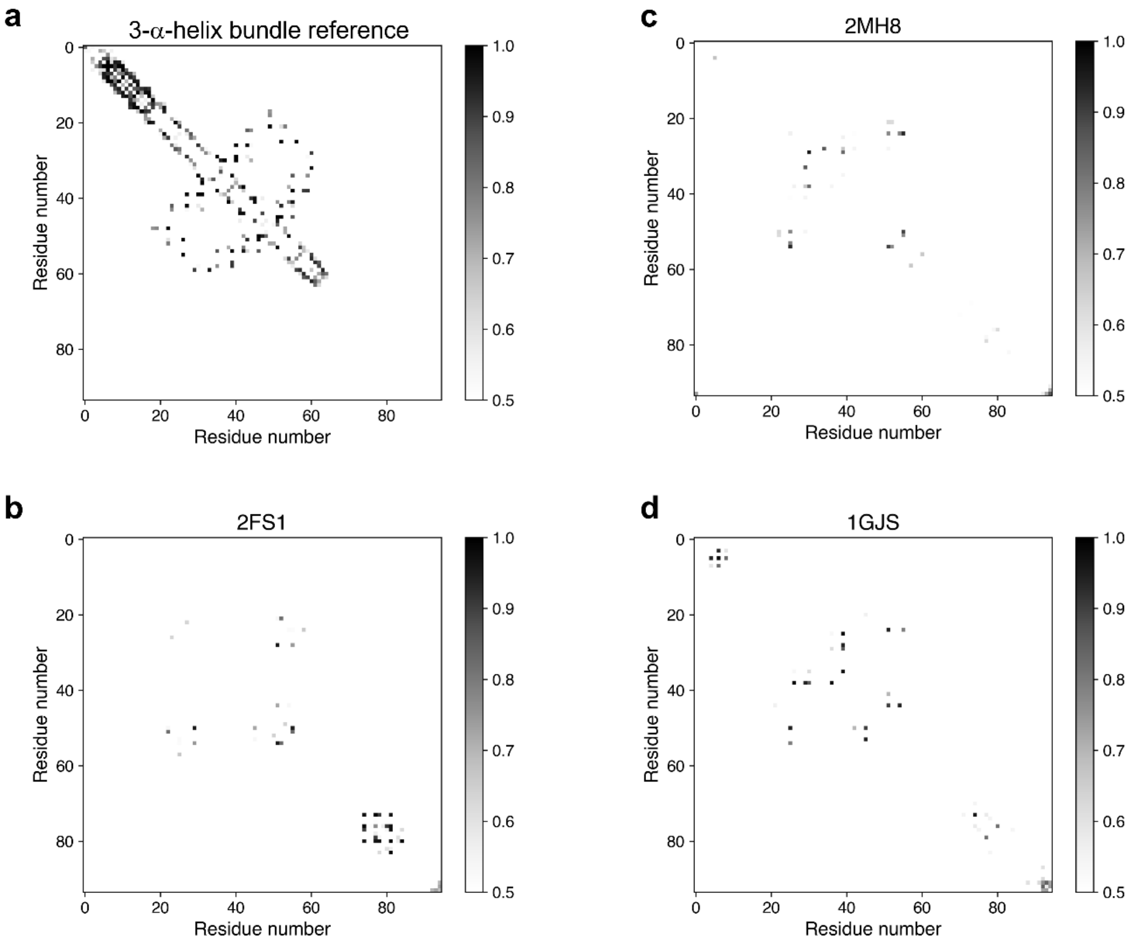

Supplementary Figure 2. Two-sequence MSAs do not contain enough evolutionary information to specify the 3-α-helix bundle fold from direct coevolutionary inference. Whereas the full MSA of a 3-α-helix bundle sequence (1GJS) contains robust evolutionary couplings consistent with its fold (a), the two-sequence MSAs that AlphaFold used to successfully predict high-confidence 3-α-helix folds contain few robust couplings (b,c,d). Titles of b,c,d correspond to PDB IDs whose sequences were used to make predictions (along with the sequence of Sa1, PDB ID: 8E6Y). Plots are contoured by contact probabilities; those >0.5 are shown. Contouring at lower levels reveals contacts inconsistent with the 3-α-helix bundle fold for all three two-sequence MSAs. Source data are included in the Source Data file.

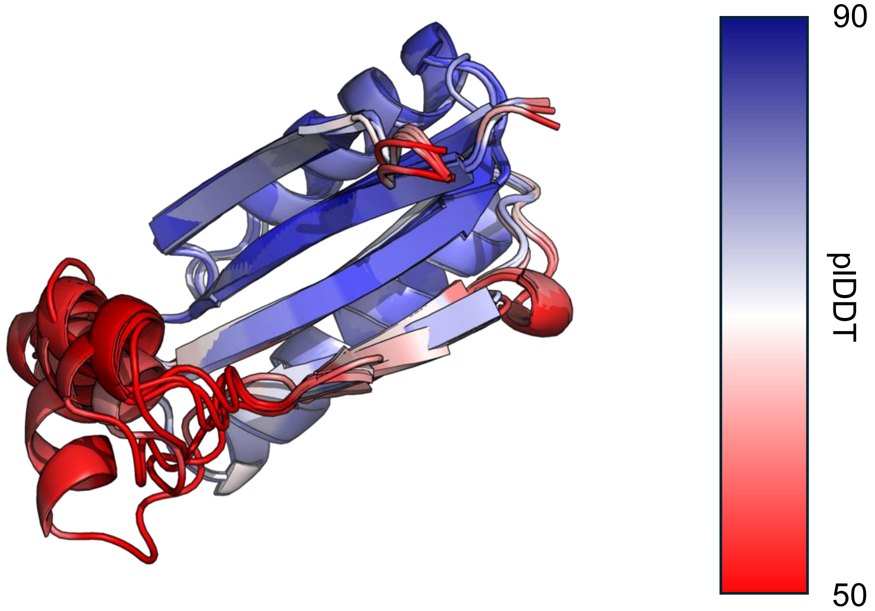

Supplementary Figure 3. AlphaFold2 predicts the fold of SA1 with high confidence from its sequence alone. This indicates that the prediction is not driven by coevolutionary inference and likely driven by sequence association; predictions were run with 3 recycles.

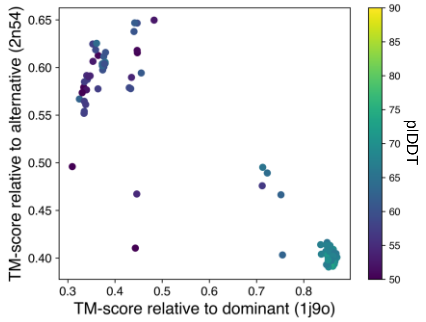

Supplementary Figure 4. AF2 can predict correct alternative conformations with low confidence. For instance, the dominant conformation of XCL1 is consistently predicted with plDDT ≥70, its alternative fold is often predicted with plDDT <70, indicating uncertain predictions. Source data are included in the Source Data file.

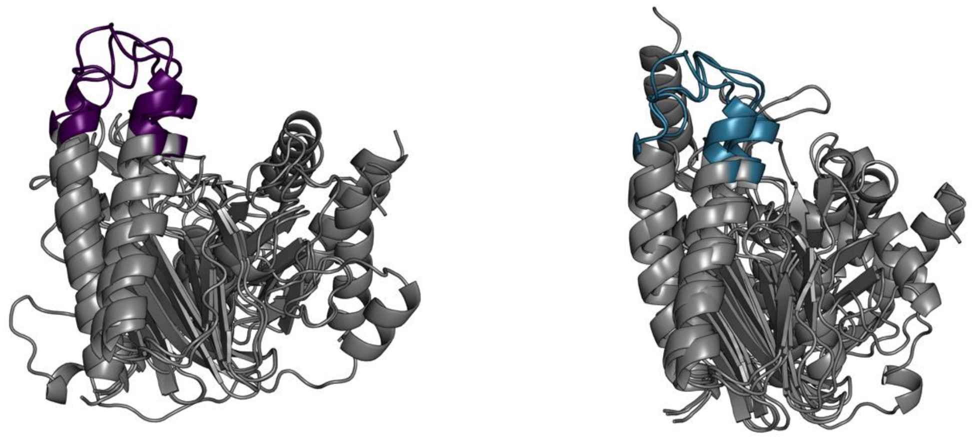

Supplementary Figure 5. CF-random correctly predicted IMPase structures from blind search in yellow and green clusters (**Figure 6**). Structures from purple and blue clusters (colored above by their respective clusters) are not as well folded as green and yellow structures.

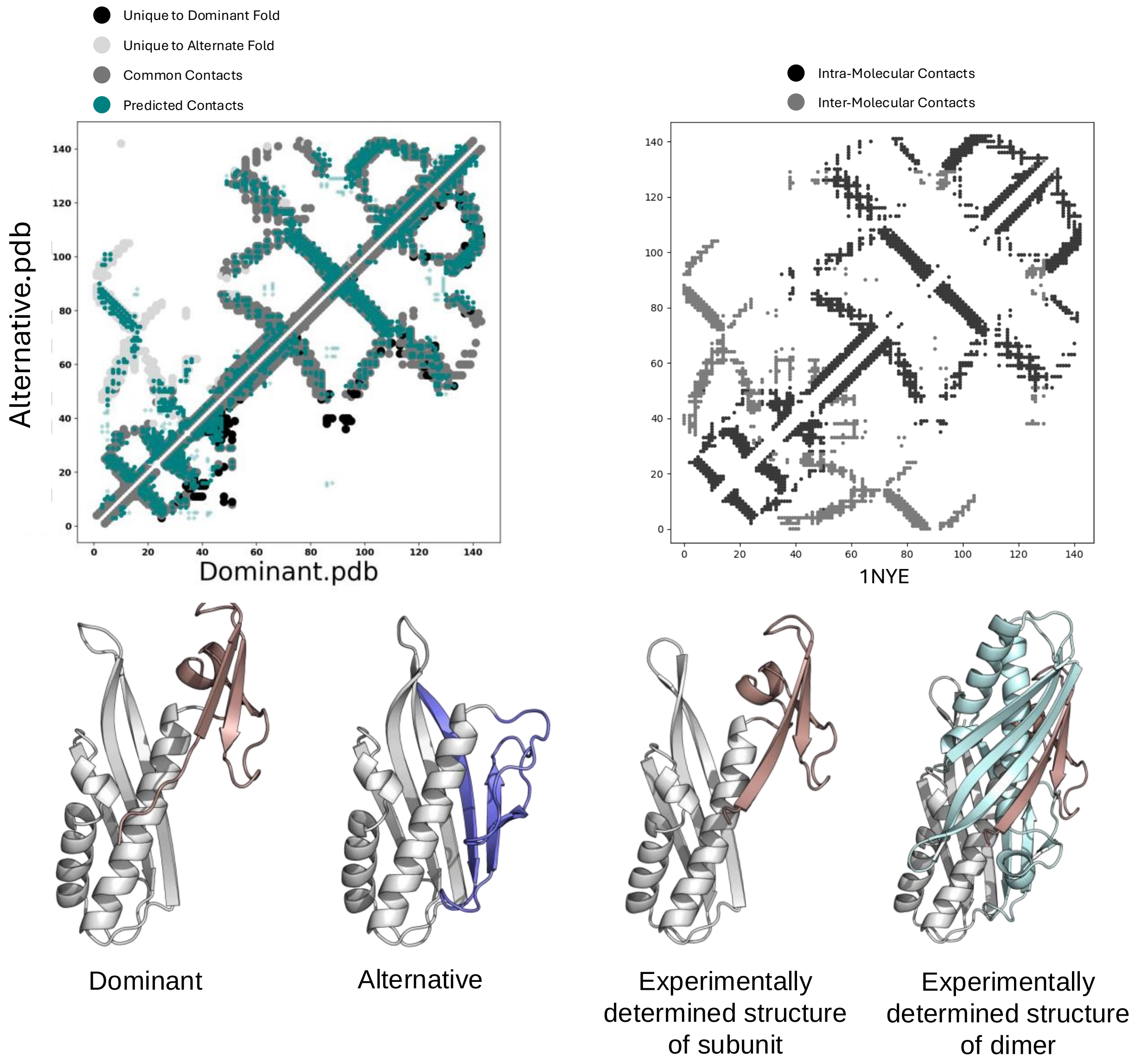

Supplementary Figure 6. CF-random sometimes conflates interchain and intrachain contacts. While the dominant predicted structure of target WP_000152305.1 matches its experimentally determined structure (PDB ID: 1NYE), the putative alternative fold appears to conflate interchain and intrachain contacts. Light gray contacts and coevolutionary information (teal) in the upper diagonal of left contact map are nearly identical to interchain contacts from the experimentally determined dimer (right). Fold switching region of Dominant/Alternative colored salmon/slate respectively. Dimeric subunit is light cyan to so that interchain contacts can be visualized. Source data are included in the Source Data file.

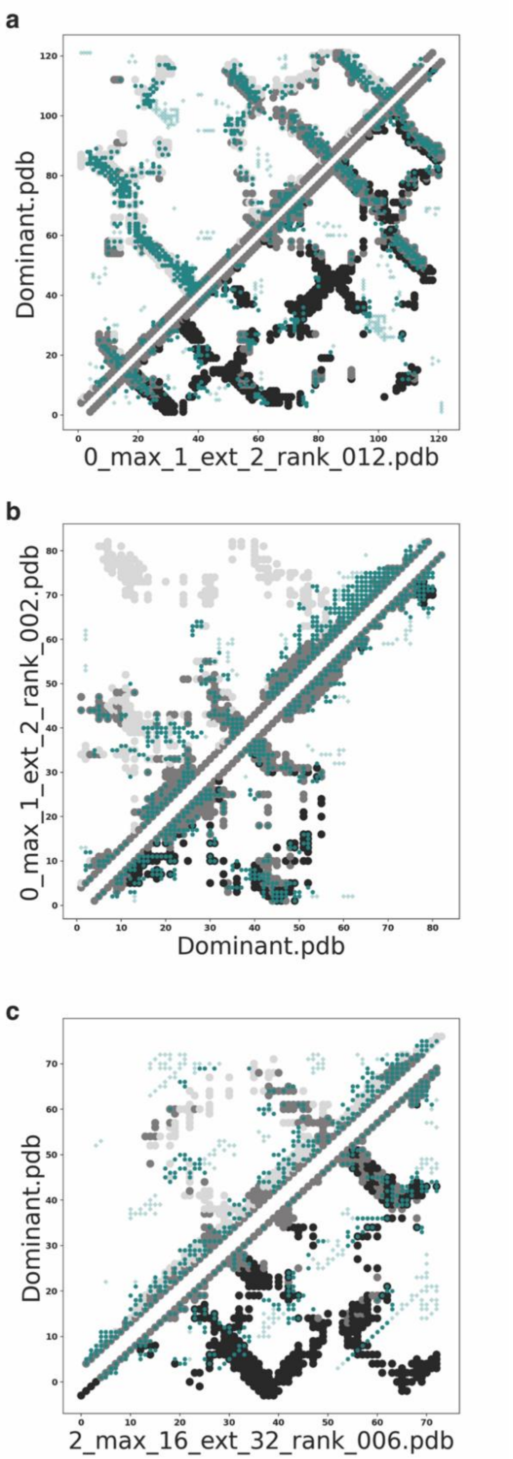

Supplementary Figure 7. Predicted fold switchers from the E. coli genome and their respective coevolutionary information. (a). Tube tail protein. (b). NinH. (c). YmcE. Dual fold contact maps are presented; the upper diagonal represents one conformation with unique contacts light gray; lower diagonal represents the alternative conformation with unique contacts black. YmcE has noisy contacts consistent with few homologs and a shallow MSA (c). Contacts common to both folds are gray; coevolutionary contacts inferred by ACE are teal. Source data are included in the Source Data file.

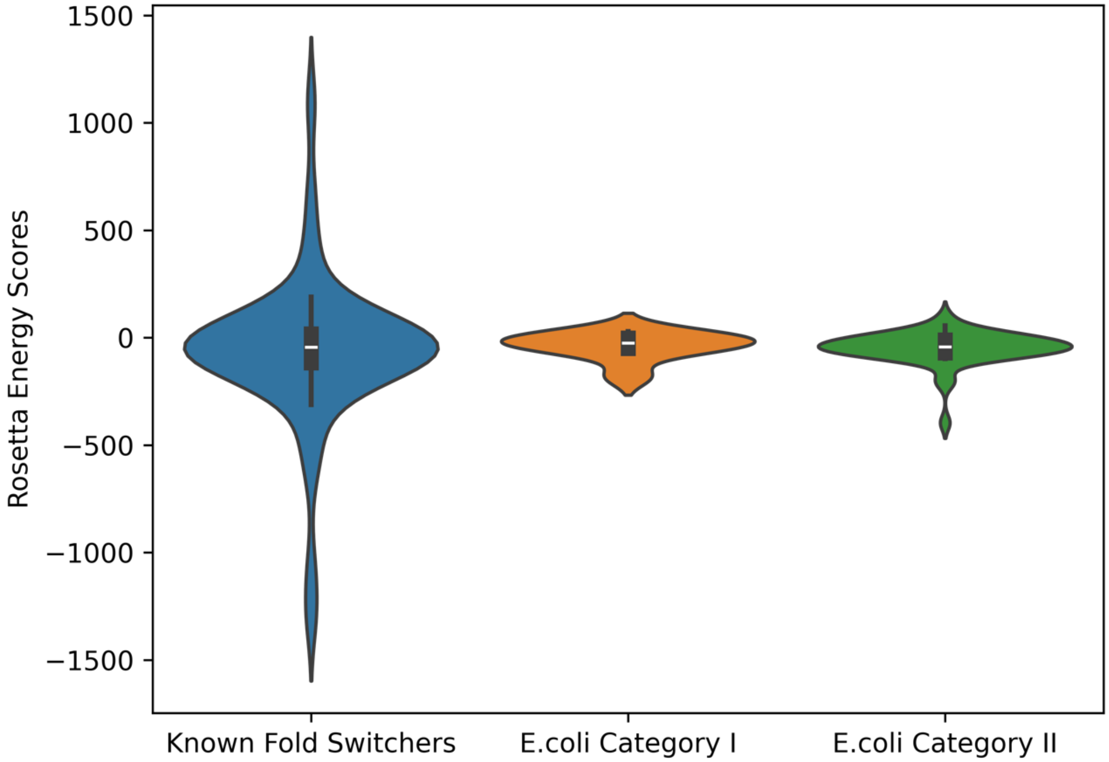

Supplementary Figure 8. Structural models of the 52 putative fold-switched pairs are plausible. The estimated difference in Rosetta energy scores between predicted models of each fold-switched pair from both Category I (with experimental and/or biological support) and Category II (experimentally unsupported) are within the same range as experimentally determined fold switchers. Source data are included in the Source Data file.

Supplementary Table 1. Sampling depths for fold-switch hits

Protein name	Fold1[1]	Depth[2]	Fold2[3]	Depth[4]
**Phytochrome**	4o0p	64:128	4o01	2:4
**α-1-antitrypsin**	1kct	16:32	3t1p	8:16
**Plasmepsin**	1qs8	16:32	1miq	32:64
**XCL1**	1j9o	512:5120	2n54	1:2
**RfaH**	6c6s_D	8:16	2oug	512:5120
**Mad2**	2vfx_A	512:5120	3gmh_L	2:4
**KaiB**	5jyt	512:5120	2qke_F	2:4
**RepE**	1rep	64:128	2z9o	2:4
**Cytolysin**	4phq	16:32	2wcd_X	1:2
**MinE**	3r9j	512:5120	2kxo	8:16
**OAS1**	4rwq	2:4	4rwn	4:8
**hTrap1**	5f5r	64:128	5f3k	4:8
**TIGR4**	4zrb_C	512:5120	4zrb_H	4:8
**Lyz**	1xjt	16:32	1xju	2:4
**IMPase**	2p3v_A	2:4	2p3v_D	2:4
**Hendra virus F protein**	5ejb	64:128	1wp8	4:8
**Nrp2**	2qqj	64:128	4qds	8:16
**CBD**	1nqd	512:5120	1nqj	1:2
**FraC**	3zwg	4:8	4tsy	2:4
**Endolysin R21**	3hdf	512:5120	3hde	2:4
**VSV-Indiana glycoprotein**	5i2s	16:32	5i2m	16:32
**α-hemolysin**	4yhd	32:64	7ahl	4:8
**KSHV protease**	2pbk	512:5120	3njq	4:8
**Complement C3**	3l5n	512:5120	2a73	512:5120
**Ebola fusion glycoprotein**	5fhc	2:4	1ebo	2:4
**Cwc2**	5lj3	64:128	3tp2	64:128
**CopK**	2lel	64:128	2k0q	8:16
**Rhomboid protease**	2lep	8:16	4hdd	1:2
**FimF**	2jmr	32:64	4j3o	4:8
**CrkL-SH3C**	2lqw	1:2	2bzy	Single sequence
**Cks1**	3qy2	512:5120	1qb3	Single sequence
**Aβ42**	1iyt	512:5120	5oqv	1:2

[1]PDB ID of dominant fold that AF2 predicts from full MSAs
[2]MSA depths that produce best model of the dominant fold; sometimes more accurate models result from shallower sampling. A:B means –max-seq:--max-extra-seq parameters in ColabFold.
[3]PDB ID of alternative folds typically not predicted from full MSA.
[4]MSA depths that produce the best model of the alternative fold. A:B means –max-seq:--max-extra-seq parameters in ColabFold.

Supplementary Table 2. Sampling depths for SPEACH_AF dataset.

**Protein name**	**Input PDB**	**Outward-facing/active**[1]	**Depth**[2]	**Inward-facing/inactive**[1]	**Depth**[2]
**MCT1**	7ckr	7ckr	2:4	7da5	64:128
**STP10**	7aar	7aaq	512:5120	7aar	4:8
**LAT1**	7dsq	7dsq	16:32	6irs	512:5120
**ZnT8**	6xpf_B	6xpf_A	512:5120	6xpf_B	64:128
**ASCT2**	7bcq	7bcq	32:64	6rvx	32:64
**CGRPR**	7knt	6uva	16:32	7knt	32:64
**PTH1R**	6nbf	6nbf	64:128	6fj3	16:32
**FZD7**	7evw	7evw	8:16	6bd4	32:64
**MurJ**	6nc9	5t77	2:4	6nc9	512:5120
**PfMATE**	6fhz	3vvn	512:5120	6fhz	8:16
**SERT**	5i6x	5i6x	512:5120	6dzz	4:8
**CCR5**	7f1q	5uiw	512:5120	7f1q	16:32
**AK**	1ake	1ake	512:5120	4ake	4:8
**RBP**	1ba2	1ba2	2:4	2dri	512:5120

[1]PDB IDs used for SPEACH-AF targets; sequences used for predictions [2]Sampling depths used to predict the opposite PDB conformation. IE, for the sequence of 1ba2, the structure is predicted from full MSA, but producing structures from that same MSA at 16:32 yields the 2dri conformation.

Supplementary Table 3. Sampling depths and PDB list of OC23 dataset.

Uniprot ID[1]	conformation – open	Depth[2]	conformation – closed	Depth[2]
**A2RJ53**	3fto_A	64:128	3drf_A	512:5120
**O76728 (reference failed)**	4bp8_A	8:16	4bp9_A	512:5120
**P31133**	6yed_B	16:32	6ye0_B	64:128
**P00558 (reference failed)**	2xe6_A	32:64	2wzd_A	8:16
**P40131**	3tee_A	16:32	3vjp_A	4:8
**Q7DAU8**	3l6g_A	16:32	3l6h_A	512:5120
**P21589**	7qga_A	16:32	4h2i_A	64:128
**A0QTT2**	7cy2_A	512:5120	7cyr_A	4:8
**Q5F9M1**	3zsf_A	512:5120	2yln_A	512:5120
**Q9X6R4**	3iuj_A	16:32	3iuq_A	512:5120
**Q18A65**	6hnj_A	32:64	6hni_A	64:128
**Q9ERE7 (reference failed)**	2rqm_A	32:64	2rqk_A	64:128
**P62495 (reference failed)**	2ktv_A	64:128	2ktu_A	8:16
**A0A075Q0W3**	6mka_A	32:64	6mkj_A	512:5120
**P71447**	2wfa_A	4:8	2wf5_A	64:128
**P33284**	3o6w_A	512:5120	3o8m_A	64:128
**Q9Z4N6**	1si1_A	4:8	1si0_A	16:32
**A6UVT1**	6hac_A	512:5120	6hae_A	512:5120
**B7IE18**	6nc7_A	512:5120	6nc6_A	32:64
**B3EYN2**	5ho2_A	32:64	5ho0_A	64:128
**Q53W80**	7c63_A	512:5120	7c66_A	512:5120
**Q9SS90**	6k8b_A	512:5120	6k85_B	512:5120
**Q9X9P9**	2olo_A	64:128	2oln_A	512:5120

[1]UniRef IDs for each AFSample2 target. “Reference failed” means AFSample2 failed to predict the alternative conformation. Red=CF-random predictions failed because the best TM-scores of both predictions were <0.7 (Q9ERE7). [2]Sampling depths used to predict the protein’s alternative conformation.

Supplementary Table 4. Putative *E. coli* fold switchers.

Protein	Function	Tier
WP_001185665	cell division regulator	I
WP_001192396	transcription / translation regulator	I
WP_001264088	transport protein / amyloid	I
WP_000064148	transcription / translation regulator	I
WP_001272149	structural assembly protein	I
WP_001119863	enzyme	I
WP_001217394	unknown	I
WP_001260507	protein targeting assembly	I
WP_000190655	enzyme	II
WP_001262174	transcription / translation regulator	II
WP_001270286	toxin-antitoxin system	II
WP_001270809	iron transport	II
WP_001272856	metal binding	II
WP_001279084	transcription / translation regulator	II
WP_001281772	replication alleviation	II
WP_001295442	motor protein	II
WP_001296140	unknown	II
WP_001296901	unknown	II
WP_001280953	toxin-antitoxin system	II
WP_001282181	amino acid transport / metabolism	II
WP_001300163	unknown	II
WP_001303590	structural assembly protein	II
WP_001316982	toxin-antitoxin system	II
WP_000015473	phage protein	II
WP_000024392	enzyme	II
WP_000134927	unknown	II
WP_000323025	toxin-antitoxin system	II
WP_000581937	toxin-antitoxin system	II
WP_000617148	unknown	II
WP_000648420	structural assembly protein	II
WP_000675390	transcription / translation regulator	II
WP_000699809	unknown	II
WP_000705622	transcription / translation regulator	II
WP_000763330	phage protein	II
WP_000803992	transcription / translation regulator	II
WP_000807125	enzyme	II
WP_000841554	transcription / translation regulator	II
WP_000847304	phage protein	II
WP_000881326	transcription / translation regulator	II
WP_000920571	transcription / translation regulator	II
WP_000951334	unknown	II
WP_000955366	unknown	II
WP_000956458	structural assembly protein	II
WP_000976004	unknown	II
WP_000994516	phage protein	II
WP_001002059	unknown	II
WP_001023459	phage protein	II
WP_001070563	unknown	II
WP_001129553	transcription / translation regulator	II
WP_001241339	unknown	II
WP_001204859	transcription / translation regulator	II
WP_001151233	unknown	II
