# Supplementary material for: Large-scale predictions of alternative protein conformations by AlphaFold2-based sequence association
Source: Nat Commun. 2025 Jul 1;16:5622. doi: 10.1038/s41467-025-60759-5 (PMC12219334; doi:10.1038/s41467-025-60759-5)
Supplement: Supplementary file 2 — Reporting Summary [file 41467_2025_60759_MOESM2_ESM.pdf]

nature portfolio

Corresponding author(s): Lauren L. Porter

Last updated by author(s): May 29, 2025

Reporting Summary

Nature Portfolio wishes to improve the reproducibility of the work that we publish. This form provides structure for consistency and transparency in reporting. For further information on Nature Portfolio policies, see our Editorial Policies and the Editorial Policy Checklist.

Statistics

For all statistical analyses, confirm that the following items are present in the figure legend, table legend, main text, or Methods section.

n/a	Confirmed	
☐	☒	The exact sample size (n) for each experimental group/condition, given as a discrete number and unit of measurement
☐	☒	A statement on whether measurements were taken from distinct samples or whether the same sample was measured repeatedly
☒	☐	The statistical test(s) used AND whether they are one- or two-sided<br>*Only common tests should be described solely by name; describe more complex techniques in the Methods section.*
☒	☐	A description of all covariates tested
☒	☐	A description of any assumptions or corrections, such as tests of normality and adjustment for multiple comparisons
☐	☒	A full description of the statistical parameters including central tendency (e.g. means) or other basic estimates (e.g. regression coefficient) AND variation (e.g. standard deviation) or associated estimates of uncertainty (e.g. confidence intervals)
☒	☐	For null hypothesis testing, the test statistic (e.g. F, t, r) with confidence intervals, effect sizes, degrees of freedom and P value noted<br>*Give P values as exact values whenever suitable.*
☒	☐	For Bayesian analysis, information on the choice of priors and Markov chain Monte Carlo settings
☒	☐	For hierarchical and complex designs, identification of the appropriate level for tests and full reporting of outcomes
☒	☐	Estimates of effect sizes (e.g. Cohen's d, Pearson's r), indicating how they were calculated

Our web collection on statistics for biologists contains articles on many of the points above.

Software and code

Policy information about availability of computer code

Data collection	ColabFold1.5.5, AlphaFold3, FoldSeek 9.427df8a, Protein BLAST webserver
Data analysis	PyMOL, TM-align, custom code written for python3 (https://github.com/mslee1220/CF-random_software), Rosetta

For manuscripts utilizing custom algorithms or software that are central to the research but not yet described in published literature, software must be made available to editors and reviewers. We strongly encourage code deposition in a community repository (e.g. GitHub). See the Nature Portfolio guidelines for submitting code & software for further information.

Data

Policy information about availability of data

All manuscripts must include a data availability statement. This statement should provide the following information, where applicable:

- Accession codes, unique identifiers, or web links for publicly available datasets
- A description of any restrictions on data availability
- For clinical datasets or third party data, please ensure that the statement adheres to our policy

Data generated for the analysis and example data to run scripts and to create figures were deposited on GitHub: https://github.com/ncbi/CF-random_software. The supporting data generated in this study are provided in the Supplementary Information and the Source Data file. The structural data used in this study were taken from the Protein Data Bank are listed below with their accession codes –

NMR structures of human XCL1: 1J90 [https://www.rcsb.org/structure/1J9O] and 2JP1 [https://www.rcsb.org/structure/2JP1]; crystal structures of human TRAP1N: 5F5R [https://www.rcsb.org/structure/5F5R] and 5F3K [https://www.rcsb.org/structure/5F3K]; crystal structures of E. coli RepE: 1REP [https://www.rcsb.org/structure/1REP] and 2Z9O [https://www.rcsb.org/structure/2Z9O]; crystal structure of FraC with lipids 4TSY: [https://www.rcsb.org/structure/4TSY]; crystal structure of the cell cycle regulatory protein Cks1 1QB3 [https://www.rcsb.org/structure/1QB3]; NMR structure of amyloid-beta fibrils: the Osaka mutations 2MVX [https://www.rcsb.org/structure/2MVX]; NMR structure of pore-forming amyloid-beta tetramers 6RHY [https://www.rcsb.org/structure/6RHY]; crystal structure of Thermotoga maritima IMPase TM1415 2P3V [https://www.rcsb.org/structure/2P3V], chains A and D; NMR structure of Sa1_V90T 8E6Y [https://www.rcsb.org/structure/8E6Y]; NMR structure of the Albumin binding domain of Streptococcal Protein G 1GJS [https://www.rcsb.org/structure/1GJS]; GA-79-MBP CS-rosetta structures 2MH8 [https://www.rcsb.org/structure/2MH8], and solution NMR structure of PSD-1 2FS1 [https://www.rcsb.org/structure/2FS1]. Unless otherwise stated, all data supporting the results of this study can be found in the article, supplement, and source data files. Source Data are provided with this paper.

Research involving human participants, their data, or biological material

Policy information about studies with human participants or human data. See also policy information about sex, gender (identity/presentation), and sexual orientation and race, ethnicity and racism.

Reporting on sex and gender	N/A
Reporting on race, ethnicity, or other socially relevant groupings	N/A
Population characteristics	N/A
Recruitment	N/A
Ethics oversight	N/A

Note that full information on the approval of the study protocol must also be provided in the manuscript.

Field-specific reporting

Please select the one below that is the best fit for your research. If you are not sure, read the appropriate sections before making your selection.

☒ Life sciences ☐ Behavioural & social sciences ☐ Ecological, evolutionary & environmental sciences

For a reference copy of the document with all sections, see nature.com/documents/nr-reporting-summary-flat.pdf

Life sciences study design

All studies must disclose on these points even when the disclosure is negative.

Sample size	92 fold-switching protein pairs, 37 other proteins that undergo conformational changes, and Sa1 (130 proteins total)
Data exclusions	Not relevant to this study
Replication	CF-random was run with 5+ random seeds to ensure robustness of results
Randomization	Not relevant to this study.
Blinding	Not relevant to this study.

Reporting for specific materials, systems and methods

We require information from authors about some types of materials, experimental systems and methods used in many studies. Here, indicate whether each material, system or method listed is relevant to your study. If you are not sure if a list item applies to your research, read the appropriate section before selecting a response.

Materials & experimental systems

n/a	Involved in the study	
☒	☐	Antibodies
☒	☐	Eukaryotic cell lines
☒	☐	Palaeontology and archaeology
☒	☐	Animals and other organisms
☒	☐	Clinical data
☒	☐	Dual use research of concern
☒	☐	Plants

Methods

n/a	Involved in the study	
☒	☐	ChIP-seq
☒	☐	Flow cytometry
☒	☐	MRI-based neuroimaging
